# Understanding barriers and facilitators to cervical cancer prevention among young Korean Women: A focus group interview study

**DOI:** 10.1371/journal.pone.0353242

**Published:** 2026-07-10

**Authors:** Hyun Jin Kim, Hyeon Ji Lee, Hye Young Shin, Hye-Sun Lee, Jae Kwan Jun, Kui Son Choi, Mina Suh

**Affiliations:** 1 National Cancer Control Institute, National Cancer Center, Goyang-si, Gyeonggi-do, South Korea; 2 Department of Nursing, Gangseo University, Seoul, Republic of Korea; 3 Department of Cancer Control and Policy, Graduate School of Cancer Science and Policy, National Cancer Center, Goyang-si, Gyeonggi-do, South Korea; Tarbiat Modares University Faculty of Medical Sciences, IRAN, ISLAMIC REPUBLIC OF

## Abstract

Cervical cancer is a largely preventable and controllable disease. Social perceptions regarding sexual experiences and gynecological visits contribute to stigma, which serves as a major barrier to screening and human papillomavirus vaccination. This study explored how stigma is formed and reinforced within sociocultural norms and personal psychological barriers. Focus group interviews were conducted with 14 Korean women aged 20–29 years, divided into screeners and non-screener groups, on November 23, 2024. Based on the transcriptions, Thematic analysis was performed on the transcripts to organize codes into core themes,(in compliance with qualitative research reporting standards). A total of 28 codes were grouped into 10 sub-themes and classified by group. Among them, three sub-themes were common to both screeners and non-screeners, including negative perceptions of gynecological visits, stigma associated with sexually transmitted diseases, and barriers due to a lack of information. Among screeners, four sub-themes were identified: a desire for alternative notification methods for screening, psychological barriers to screening, a sense of burden from additional tests during visits, and reluctance to share screening experiences due to perceived prejudice from others. Among non-screeners, three sub-themes were identified: stigma toward patients with cervical cancer and lack of awareness of the sensitivity of cervical cancer. To promote cervical cancer preventive behaviors, stigma must be addressed as a social process rather than solely an individual issue. Practical solutions include creating a predictable screening environment (e.g., prior notification of procedures and additional tests with consent), improving patient-centered care, and enhancing notification methods. This study provides a foundation for developing stigma-related indicators and designing follow-up studies. (e.g., individual interviews/large-scale surveys).

## Introduction

Cervical cancer is the leading female genital tract cancer caused by persistent human papillomavirus(HPV) infection. [1] As of 2022, it remains a significant global public health issue, with approximately 660,000 new cases and over 350,000 deaths worldwide. [2,3] The World Health Organization (WHO) has classified cervical cancer as a “preventable cancer” and, announced a global strategy for its elimination in 2020. [4,5] This strategy aims to achieve a 90% HPV vaccination rate among girls aged under 15 years, a 70% screening rate for women aged 35–45 years, and a 90% treatment rate for high-risk women by 2030. [6] Globally, the HPV vaccine has been included in national immunization programs in more than 140 countries, and along with expanded vaccine access, the adoption of single-dose vaccinations is spreading. [7]

In Korea, cervical cancer prevention policies have been consistently pursued through the National Cancer Screening Program (NCSP) and the National Immunization Program (NIP). The NCSP, implemented in 2002, initially targeted women aged 30 years and above, but the starting age was lowered to 20 years in 2016 in response to the increasing incidence of cervical cancer among younger women. Under this program, women aged 20 years and above are offered free Pap smear tests every 2 years. [8] In addition, since 2016, the National NIP has provided free human papillomavirus (HPV) vaccination for 12-year-old girls to strengthen primary prevention efforts. [9] Consequently, the overall incidence and mortality rates of cervical cancer have shown a downward trend. However, recent data indicate a significant decrease in screening rates in women aged 30–74 years. [10] However, the cervical cancer screening rate among Korean women in their 20s remains low at 45.8% as of 2022, substantially below WHO’s recommended 70–90% coverage target for women aged 35–45 years in cervical cancer elimination strategies (World Health Organization, 2022)[Organization, 2022 #91]; [11,12] the current HPV vaccination rate among adolescent girls is approximately 75%.

To understand trends in prevention behaviors, existing domestic studies have primarily focused on quantitative and qualitative statistical data regarding regular screening compliance, vaccination expansion scenarios, and changes in screening and vaccination. [13,14] According to previous studies, participation in cervical cancer screening is greatly influenced by individual knowledge and attitudes and by social norms and cultural perceptions. An analysis of perceptions and participation barriers among Korean female university students in their 20s confirmed that sociocultural factors such as conservative sexual culture, negative perceptions of the parents’ generation, and stigma about sexual experience are major deterrents to screening participation. [15] Qualitative studies on psycho-social factors that affect cervical cancer prevention behaviors are already being conducted overseas, [16] and the need for a qualitative approach is increasingly emphasized in Korea. [17]

Recent international literature has increasingly highlighted the multilevel nature of barriers to cervical cancer prevention, extending beyond individual knowledge deficits to encompass structural, sociocultural, and stigma-related dimensions. A 2025 systematic review of studies published through 2024 identified fear, embarrassment, cultural and religious norms, and distrust in healthcare systems as recurring barriers across diverse populations, with qualitative approaches being particularly valuable in capturing these nuanced experiences. [18] Furthermore, a 2025 systematic review focusing specifically on young women confirmed a global decline in screening and HPV vaccination uptake in this age group, underscoring the urgent need for targeted, context-sensitive interventions. [19] These trends point to the limitations of knowledge-focused approaches alone and call for a deeper examination of the psychosocial and sociocultural mechanisms that shape prevention behavior. In particular, stigma should be understood as a complex process that is constructed and reproduced within social relationships, rather than merely as an attribute of an individual. [20,21]

In 1963, Goffman defined stigma as a “discrediting attribute.” However, subsequent studies in the context of HIV/AIDS have shown that stigma is not a fixed personal trait but a social process that changes and resists according to cultural and social contexts. [22,23] This suggests that for diseases closely linked to sexual experience, such as cervical cancer, stigma should be examined within sociocultural norms and institutional environments rather than reduced to individual morality. [23–25]

Accordingly, in this study, we explored perceptions and stigma factors among Korean women in their 20s, encompassing all cervical cancer prevention behaviors, especially focusing on screening, HPV vaccination, and sexual health management. We specifically sought to identify the impact of sex-related stigma, reluctance toward gynecological visits, and sociocultural prejudice on participation in screening, which has not been sufficiently highlighted in previous studies. Overall, we aimed to comprehensively understand the structural and psychological barriers that hinder prevention behaviors and provide foundational data for developing stigma-related indicators of cervical cancer. This qualitative study employed focus group interviews (FGIs) to analyze the sociocultural factors related to cervical cancer screening participation, offering practical implications for promoting preventive behaviors among young Korean women.

## Materials and methods

### Study design

This study employed a qualitative research approach to deeply explore potential facilitators and barriers to cervical cancer screening as well as perceptions about cervical cancer, cervical cancer screening, HPV, and HPV vaccine among Korean women in their 20s.

FGIs were conducted with open-ended semi-structured questions to investigate their perceptions. The research process and results are reported using the Consolidated Criteria for Reporting Qualitative Research. [26,27].

### Focus group participants and setting

The study participants were Korean women aged 20–29 years who agreed to participate in FGIs. They were divided based on their cervical cancer screening history, with two FGIs conducted, one for each group. Participants were recruited through snowball sampling using promotional posters and an online application (Google Forms). According to the recommendation that FGIs should include a minimum of 4 and a maximum of 12 participants; [27] this study included 14 participants, divided into a screener group (Group A) of 4 and a non-screener group (Group B) of 10. Those who were unreachable after confirmation were considered to have no intention to participate and were thus excluded (Group A, n = 1).

### Data collection

The interviews were conducted on Saturday, November 23, 2024. The screener group (Group A) interview was held at 1 p.m., and the non-screener group (Group B) interview was held at 4 p.m., with each session lasting approximately 2 h. The FGIs were conducted in an office specializing in interviews and were video recorded on an online platform with restricted access. The interviews were conducted face-to-face by a female professional facilitator from an external organization, who had prior experience conducting FGIs relating to the HPV vaccine. Based on a related study, [15] the research team structured the questionnaire and interview guide (Table 1). A thorough discussion with the facilitator was held beforehand to ensure adherence to the study protocol. During the interviews, related videos and materials were provided to question participants about the changes in their perceptions before and after acquiring new information. The research team and facilitator had no prior contact with the participants.

**Table 1:** Focus group interview outline.

Stage of interview	Summary of Questions
Opening	Warming up Introduction to the purpose of the study, self-introduction of the research participants
Part 1. Perceptions of cervical cancer	**(Consensus)** What do you know about cervical cancer? What is your image of a patient with cervical cancer? Who do you think is more likely to develop cervical cancer? (e.g., age, personality, lifestyle) Do you think patients with cervical cancer are responsible for their illness?
Part 2. Perceptions of cervical cancer screening	**(Consensus)** What do you think about the idea that you can only get a cervical cancer screening if you had sexual experience? **(Screeners)** Have you ever shared your cervical cancer screening experience with those around you (e.g., boyfriend/family/friends)? If not, why? Are you uncomfortable with revealing your sexual experience during a cervical cancer screening? **(Non-screeners)** How do you feel about the prospect of getting a cervical cancer screening? Would you share your cervical cancer screening with those around you? ‘How did you feel when you heard that “if you don’t have sexual experience, you don’t need to get a screening”’?
* Perceptions and attitudes about cervical cancer screening were **interviewed both before and after watching** related materials and videos.	
Closing	Has your opinion changed through this interview?

### Data analysis

The participants’ general characteristics were summarized as descriptive statistics based on their self-reported responses during the interviews. All interview content was anonymized and transcribed using audio and video recordings. The co-first authors (HJL and HJK) performed thematic analysis based on the transcripts. [28,29] Repeated concepts were extracted through repeated reviews of the transcripts, and sentences and keywords related to the questions in each interview were labeled as codes. The codes were organized into subcategories and categories. Transcript reviews continued until no new codes emerged. Transcripts were coded using the open-source tool Taguette (https://app.taguette.org/ (accessed on July 14, 2025). [30]

### Ethical consideration

This study received Institutional Review Board approval from the National Cancer Center (approval no.: NCC2024−0254). The recruitment notice and application form specified the research topic and key content, research institution and team, research method (FGI format, duration, and location), and items for personal information collection and consent. All participants were fully informed of the study’s purpose and procedures before the interviews and voluntarily signed a written consent form if they wished to participate. All interviews were audio- and video-recorded with prior consent, and anonymity and personal information were strictly protected throughout the research process.

## Results

### Focus group characteristics

Table 2 presents the characteristics of the participants by group, based on their self-reported responses during the interviews and self-introductions, categorized by their cervical cancer screening history. Of the 14 participants, 4 had prior cervical cancer screening experience, whereas 10 had none. The average age of the screening group was 24.0 years (SD = 3.37), whereas that of the non-screening group was 21.5 years (SD = 1.84), making the non-screening group relatively younger. In Group A, all but one participant completed the HPV vaccination, whereas in Group B, the proportion of those who had not been vaccinated was higher. Some individuals in Groups A and B had gynecological diseases. Most participants in both groups did not smoke, and all but one participant who refused to answer stated that they consumed alcohol, although the frequency and amount varied.

**Table 2:** Characteristics of the focus group interviews.

Interview	Focus group A (Cervical cancer screener)	Focus group B (Cervical cancer non-screener)
**Number of participants (N)**	4	10
**Age**		
Range	23-29	19-25
Mean (SD)	24 (3.37)	21.5 (1.84)
Median (IQR)	22.5 (22-24.5)	22 (20.3-22)
**HPV vaccination status**		
Yes	3	4
No	1	6
**Gynecology disease experience**		
Yes	2	6
No	2	3
No response	0	1
**Current smoking status**		
Yes	1	1
No	3	8
No response	0	1
**Current alcohol drinking status**		
Yes	4	9
No	0	0
No response	0	1

### Qualitative analysis

A total of 28 codes were derived from the interviews and organized into 10 subthemes. Quotes and participants’ responses to the core themes were also presented. “M” was used for topics agreed upon by most, “S” for topics agreed upon by some, and “N” when a group did not agree on the content, or it was not mentioned due to group differences (Table 3). For clear identification, each quotation included the participant’s number and basic information (group, age, and gynecological disease history).

**Table 3:** Factors influencing cervical cancer prevention behaviors (N = 14).

Groups	Category	Quotes	A (N = 4)	B (N = 10)
Consensus	1) Negative perception of gynecological visits	“First of all, I think the perception of gynecological clinics needs to change. When a young woman goes alone, the people around her give her a bad look∙∙∙” “When I go to a gynecological clinic, if I run into a friend from the same high school, I feel like I need to explain why I’m there. It’s unnecessary feeling, but it just comes up.”	S	M
	2) Stigma as a sexually transmitted disease – Burden of revealing sexual experience	“I feel awkward saying that I have sexual experience.” “Even though I now know it’s not true, I couldn’t bring myself to tell those people. They already had that perception, and I knew they would see me the same way.”	M	S
	3) Barriers due to the lack of information	“I’m not sure if (HPV) is 100% transmitted through sexual contact.”	N	S
Screeners	4) Desire for alternative delivery methods besides existing ones	“It comes by mail, but if the address changes, I can’t receive it. So, it would be great if it came as a KakaoTalk message.” “If comes by mail, but if the address changes, I can’t receive it. So, it would be great if it came as a KakaoTalk message.”	S	N
	5) Psychological barriers to screening	“It’s because of the discomfort of the instrument going in, it’s stiff and cold∙∙∙”	M	S
	6) Burden of additional tests during a visit	“My friend said she felt very uncomfortable. Suddenly this test was added, and that test was added, and it took a long time, so she felt even more uncomfortable∙∙∙”	S	N
	7) Reluctance to share screening experiences due to perceived prejudice from others	“I couldn’t tell my acquaintances. I felt like they would look at me negatively because they already have that (negative) perception.” “I need to first gauge how open-minded someone is before I can bring it up. It’s a sensitive topic-you have to know in advance how much they can handle.”	S	S
Non-screeners	8) Stigma against patients with cervical cancer	“Honestly, I don’t think there’s a woman or man who would consent to having sex with someone who says they have HPV. So, they have the image of being a dishonest person.”	M	S
	9) Low perceived risk of getting cervical cancer	“In the case of teenagers, they think it’s not their problem, and sexual experience is a future thing, so they don’t feel the seriousness of it often∙∙∙” “If someone got infected through a partner, they probably hid it. Because honestly, nobody would agree to have sex with someone who tells them upfront they have HPV.”	M	N
	10) Lack of awareness of the severity of cervical cancer	“I think I’ve thought of it as a little less serious than cancers in other organs.”	N	S

M, the subtheme emerged in most of the participants; S, the subtheme emerged in some of the participants; N, the subtheme emerged in none of the participants

### Category 1: Social stigma and information gap affecting cervical cancer screening

Common opinions from both the screener and non-screener groups were derived from three subthemes: negative perception of gynecological visits, stigma of being a sexually transmitted disease, and barriers due to lack of information. The screener group showed pronounced social stigma regarding gynecological visits and stigma related to sexual experiences. Specifically, participants noted that visiting a gynecological clinic is linked to social judgment, which lowers young women’s access to healthcare institutions.

*“First of all, I think the perception of gynecological clinics needs to change. When a young woman goes alone, the people around her give her a bad look...”* (Screener D, born ‘99, with a gynecological disease).

*“I feel awkward saying that I have sexual experience.”* (Screener B, born ‘02, no gynecological disease).

Although all participants had a basic understanding that cervical cancer and HPV are sexually transmitted, they lacked accurate information sources and detailed knowledge. Several participants were uncertain about fundamental facts regarding cervical cancer. In the absence of reliable knowledge, all sexually transmitted diseases were often assumed to result from promiscuous behavior. The frequent media portrayal of young women visiting gynecological clinics alone and the spread of misinformation by acquaintances are thought to have created a social atmosphere in which discussing cervical cancer and its prevention is taboo.

*“I’m not sure if (HPV) is 100% transmitted through sexual contact.”* (Non-screener A, born ‘02, with a gynecological disease).

### Category 2: Psychological and environmental barriers that make cervical cancer screening difficult

Four subthemes were derived from the screener group: desire for alternative notification methods, psychological barriers to screening, burden of additional tests, and reluctance to share screening experiences owing to perceived prejudice. Some participants expressed that it would be beneficial to receive screening notifications via text messages without having to look for them. There was also a preference for notifications via KakaoTalk, the most widely used mobile messaging application in South Korea, which were checked more frequently than email which could be lost.

*“It comes by mail, but if the address changes, I can’t receive it. So, it would be great if it came as a KakaoTalk message.”* (Screener C, born ‘95, no gynecological disease).

Some participants also expressed discomfort with gynecological screening and the burden of unexpected additional tests. When asked what they thought about the screening, two out of four participants described it as “uncomfortable” and “scary,” and felt the process was physically unpleasant. Some respondents indicated that unexpected requests for additional tests made the screening experience negative. As a negative screening experience can lower the willingness to re-participate, a predictable and trustworthy screening environment must be created throughout the entire process.

*“It’s because of the discomfort of the instrument going in, it’s stiff and cold...”* (Screener C, born ‘95, no gynecological disease).

*“My friend said she felt very uncomfortable. Suddenly, this test was added, and that test was added, and it took a long time, so she felt even more uncomfortable...”* (Screener B, born ’02, no gynecological disease).

Even after screening, many were reluctant to share the fact that they had been screened, indicating the existence of social stigma.

*“I couldn’t tell my acquaintances. I felt like they would look at me negatively because they already have that (negative) perception.”* (Screener D, born ’99, with a gynecological disease).

### Category 3: Low perceived benefit versus dual psychological and physical barriers

Three subthemes were derived from the non-screener group: stigma toward patients with cervical cancer, low perceived risk of the disease, and lack of awareness of its severity. In this topic, questions about patients with cervical cancer revealed stigma of sexually transmitted diseases, with some thinking that patients were responsible for their illnesses. Although there was a lack of awareness regarding cervical cancer, watching a video of a real patient’s case increased perceived threat. As such educational methods can motivate preventive behaviors by changing perceptions in a short time, the need to expand this type of education is apparent.

*“Honestly, I don’t think there’s a woman or man who would consent to having sex with someone who says they have HPV. So, they have the image of being a dishonest person.” (Non-screener B, born ‘02, with a gynecological disease)*.

Participants generally did not perceive cervical cancer as a serious disease and tended to view it as less severe than other types of cancer. After watching an educational video, opinions changed, with participants saying things like, “I thought it was a more serious disease than I realized,” and “I became more aware of it”.

*“In the case of teenagers, they think it’s not their problem, and sexual experience is a future thing, so they don’t feel the seriousness of it often...”* (Non-screener E, born ‘05, no gynecological disease).

*“I think I’ve thought of it as a little less serious than cancers in other organs.”* (Non-screener D, born ‘02, with a gynecological disease).

## Discussion

This study used FGIs to explore the perceptions of cervical cancer and identified sociocultural factors that influence prevention behaviors among young Korean women in their 20s. The analysis highlighted three main barriers to cervical cancer screening: stigma related to sexual experiences, negative perceptions of gynecological visits, and psychological or environmental discomfort. [15] This confirms that stigma should not be reduced to individual morality but viewed as a socially constructed process reinforced within cultural perceptions and norms. In addition, health-related stigma operates on multiple levels—individual, organizational, and policy—and such structural stigma exacerbates health inequalities by hindering preventive behaviors and access to healthcare. [20] Particularly, the social perception that cervical cancer and HPV infection are linked to sexual experiences is a major psychological burden on screening and vaccination. [15]

Some participants misunderstood cervical cancer as a “disease for promiscuous people,” reflecting the impact of stigma in preventing them from even talking about it when they experience the disease themselves. [31] Similarly, an overseas qualitative study on breast cancer screening reported that providing personalized risk information can increase the willingness to screen, but a high-risk diagnosis can be accompanied by feelings of stigma. [32] These perceptions were also linked to conservative sexual values, especially from fathers, and the fear of screening results being discovered sometimes led to the avoidance of screening. This can be understood in a similar context to the “closed-minded view of unmarried women’s sexual health” reported in previous studies. [33]

Lack of accurate information also emerged as a major barrier. Although most participants had a basic understanding of HPV and cervical cancer, they often confused or misunderstood specific information, such as vaccination schedules, required doses, and screening cycles. The lack of sexual health education in high schools has been identified as the background for this information gap. Therefore, sexual health education for young women must be reorganized to provide more specific and practical information. Similarly, Sekhar et al. (2024) highlighted that providing accurate and reliable information is a critical component of sexuality education, as it helps to correct misinformation and misconceptions stemming from stigma and social taboos. [34]

The financial burden was also a major factor that made vaccination difficult. A previous study confirmed that the HPV vaccination rate for women in their 20s was only 38.6% in 2016, and the main reasons for non-vaccination were cost (24.3%) and concerns regarding vaccine safety (23.1%). [35] As the vaccine requires three doses, the cumulative cost often discourages uptake, and students without a fixed income often postpone or give up on the decision to get vaccinated. Furthermore, the pricing disparity between public health centers and private clinics in Korea may further complicate vaccine accessibility; women who are ineligible for the national immunization program are often redirected to private clinics where out-of-pocket costs are substantially higher, creating a two-tiered system in which access to prevention is inequitably distributed by income. One participant noted that *“If the free vaccination age were extended to women in their 20s, the vaccination rate would increase.”* This suggests that expanding vaccine policies and improving accessibility can lead to practical increases in prevention rates. The response that they postponed screenings owing to a lack of time from school or work was common, regardless of whether they had screening experience. This shows that it is not just a problem of lack of awareness, but that realistic constraints and low accessibility make it difficult for women to practice health behaviors.

In addition, recurring everyday barriers were repeatedly raised, including difficulties in accessing gynecological services, feeling self-conscious when visiting hospitals, discomfort with male medical staff, and lack of time.

Women who have experienced cervical cancer screening noted that *“medical staff should be kinder,”* highlighting how a clinical environment that does not guarantee psychological safety continuously reinforces feelings of stigma. Cervical cancer stigma operates at multiple levels, including individual shame, self-blame, social isolation, family conflicts, and community norms. [31,36] It acts as a barrier throughout prevention, screening, and treatment. [37] Participants proposed practical alternatives to alleviate these barriers, such as online information delivery, expanded support, and the introduction of a screening system that ensures anonymity.

This study has some limitations. It is a small-scale qualitative study that explored the perceptions of Korean women in their 20s and does not represent the entire population. Specifically, information on sexual experiences was not collected from all the participants, and the small number of participants in the screening group was a limitation. This can be interpreted as difficulty in recruitment due to the low screening rate among women in their 20s and the sensitivity of disclosing sexual experiences. Additionally, formal member checking such as returning summarized finding to participants for post-interview validation was not conducted in this study. Although real-time clarification and summary confirmation were performed by the moderator through each session to ensure accurate interpretation of participants’ responses, the absence of post-hoc member checking may be considered a limitation with respect to the confirmability of the findings. Nevertheless, the significance of this study lies in its ability to demonstrate specifically how stigma operates within its sociocultural context, which has not been revealed in previous quantitative surveys. It captured shared perceptions among participants and the subtle attitudes and emotions that emerged during the interaction, which were difficult to obtain through individual in-depth interviews alone. Previous overseas studies also noted that participants were intimidated by sensitive topics related to sex; however, FGI was presented as a methodology that could alleviate these barriers through group support and interaction. [16] During the group discussions, participants shared their discomfort with sex-related stigma or gynecological visit experiences, which were difficult to disclose voluntarily. This process provides important insights for a deeper understanding of the sociocultural context in which stigma is formed and reinforced, beyond simply collecting individual experiences. This study can inform the systematic design of future prevention policies, including the implementation of neutral and moral sexual health education, [34] the creation of a friendly and comfortable clinical environment, and the expansion of prevention policies for men. A large-scale meta-analysis confirmed that expanding HPV vaccination to men significantly reduces overall HPV infection rates and improves herd immunity compared to policies targeting only women. [38,39] Importantly, including men in HPV prevention campaigns may also serve to de-gender the stigma surrounding cervical cancer. As reflected in the experience of participants in this study, much of the shame and reluctance associated with screening stems from the perception is framed as a shared responsibility across genders rather than a female-specific burden, this may reduce the moral judgment directed at women and lower the psychological barriers to screening and vaccination identified in our data. These findings support the development of stigma-related indicators and large-scale follow-up studies.

## Conclusions

This study showed that the factors hindering cervical cancer prevention behaviors among young Korean women in their 20s are rooted in psychological and environmental barriers stemming from sex-related stigma, information gaps, and low satisfaction with the screening experience (e.g., unpredictability and discomfort). This qualitative study deepened the understanding of how stigma surrounding sexual experience and gynecological visits is embedded in Korean sociocultural norms. The findings highlight that stigma reduction should be considered not only at the individual level but also within institutional and cultural contexts.

Based on these insights, future cervical cancer prevention policies should aim to improve the predictability and psychological safety of screening environments, strengthen sexuality education that provides accurate information, and enhance accessibility to HPV vaccination. In particular, transitioning from mail-based screening notifications to direct digital channels such as KakaoTalk or text messaging may serve as a practical and cost-effective strategy to improve screening reach among young women, for whom postal notifications are often missed due to frequent address changes or family interception. Further research is needed to develop standardized tools for measuring stigma and to explore practical intervention models that can effectively reduce stigma-related barriers in cervical cancer prevention.
