# Supplementary material for: Understanding barriers and facilitators to cervical cancer prevention among young Korean Women: A focus group interview study
Source: PLoS One. 2026 Jul 10;21(7):e0353242. doi: 10.1371/journal.pone.0353242 (PMC13353974; doi:10.1371/journal.pone.0353242)
Supplement: Attachment — Submitted filename: Response to Reviwers.docx [file pone.0353242.s001.docx]

**Reviewer 1’s Comments:**

**Revier#1**

**# Introduction : A paragraph should not be less than 5 lines.**

Response : Thank you for this suggestion. We have revised the introduction to ensure that each paragraph meets the minimum length requirement. Specifically, the first paragraph, which previously ended after over 350,000 death worldwide, has been merged with the subsequent sentence describing the WHO classification of cervical cancer as a ‘preventable cancer’, forming a single cohesive paragraph of sufficient length. The logical flow and contextual clarity of the introduction have been maintained throughout this revision.

**#The epidemiology is old. It must be for the last two years.**

Response : We appreciate the reviewer's critical comment. In response, we have updated the epidemiological references in the Introduction to reflect the most current available data. Specifically, the following changes were made in the revised manuscript:

1. Cervical cancer screening trends in Korea: The reference for screening rate data (previously Shin et al., 2022, covering data through 2020) has been supplemented with Kang et al. (2025), which reports findings from the National Cancer Screening Survey covering data through 2023 [Reference 12 in the revised manuscript].
2. Screening rate data for women aged 30–74: Updated to include Sabatino et al. (2025) [Reference 10], providing the most current cancer screening use data.
3. Multilevel barriers to cervical cancer prevention: Two systematic reviews published in 2025 have been newly added to strengthen the epidemiological and literature context:
   - Farajimakin (2024): A systematic review of barriers to cervical cancer screening published in *Cureus* [Reference 18].
   - Shpendi et al. (2025): A systematic review identifying key barriers, facilitators, and factors associated with cervical cancer screening attendance in young women, published in *Women's Health* [Reference 19].

These updates ensure that the Introduction reflects current epidemiological evidence regarding cervical cancer screening trends and prevention barriers, with all newly added references published within the past two years (2024–2025).

**# On line 62, the source must be cited.**

Response : Thank you for this observation. A citation has been added at line 62 to support the statement regarding HPV vaccine inclusion in national immunization programs in more than 140 countries. The reference added is: *Han J, Zhang L, Chen Y, Zhang Y, Wang L, Cai R, et al. Global HPV vaccination programs and coverage rates: a systematic review. EClinicalMedicine. 2025;84:103290.*

**# On line 68, after the years, the source must be cited.**

Response : Thank you for this observation. A citation has been added following the mention of the year 2016, in reference to the establishment of the National Cancer Screening Program’s expanded coverage. The reference added is: *Lee K, Suh M, Choi KS. Current Status of the National Cancer Screening Program in Korea: History, Achievements, and Future Directions. J Prev Med Public Health. 2025;58(4):337-47.*[Reference 8 in the revised manuscript].

**# On line 69, after the efforts, the source must be cited.**

Response : Thank you for this observation. A citation has been added after “primary prevention efforts” to support the description of the National Immunization Program providing free HPV vaccination for 12-year-old girls since 2016.

**#The introduction should include a background of recent research**

Response : We thank the reviewer for this suggestion. We have expanded the introduction to include recent literature on barriers to cervical cancer prevention and screening behaviors among young women. Specifically, the following content was added after the paragraph on qualitative approaches(following line 82 of the original manuscript) : “Recent international literature has increasingly highlighted the multilevel nature of barriers to cervical cancer prevention, extending beyond individual knowledge deficits to encompass structural, sociocultural, and stigma-related dimensions. A 2025 systematic review of studies published through 2024 identified fear, embarrassment, cultural and religious norms, and distrust in healthcare systems as recurring barriers across diverse populations, with qualitative approaches being particularly valuable in capturing these nuanced experiences.[18] Furthermore, a 2025 systematic review focusing specifically on young women confirmed a global decline in screening and HPV vaccination uptake in this age group, underscoring the urgent need for targeted, context-sensitive interventions.[19]”

**# Method : Who do you think is likely to get cervical cancer? / This sentence should be corrected.**

Response : Thank you for identifying this issue. The question in the interview guide(Table1) has been revised from “Who do you think is likely to get cervical cancer?” to Who do you think is more likely to develop cervical cancer? to improve clarity and reduce the potential for stigmatizing implications in the phrasing.

**# Results : More quotes should be provided in the findings section. Because there is a need for more evidence for subthemes for the readers.**

Response : We appreciate this helpful suggestion. Additional participant quotations have been included to strengthen the evidence for each subtheme. The specific additions are as follows:

- **Subtheme 1 (Negative perception of gynecological visits):** Added quote from Screener A: *"When I go to a gynecological clinic, if I run into a friend from the same high school, I feel like I need to explain why I'm there. It’s an unnecessary feeling, but it just comes up."*
- **Subtheme 2 (Stigma as STD – Burden of revealing sexual experience):** Added quote from Screener D: *"Even though I now know it's not true, I couldn't bring myself to tell those people. They already had that perception, and I knew they would see me the same way."*
- **Subtheme 4 (Desire for alternative delivery methods besides existing ones):** Added quote from Screener C: *“*If it comes by mail, but if the address changes, I can’t receive it. So, it would be great if it came as a KakaoTalk message.”
- **Subtheme 7 (Reluctance to share screening experiences due to perceived prejudice from others):** Added quote from Screener D: “I need to first gauge how open-minded someone is before I can bring it up. It’s a sensitive topic-you have to know in advance how much they can handle.”
- **Subtheme 9 (Low perceived risk of getting cervical cancer):** Added quote from Non-screener B: “If someone got infected through a partner, they probably hid it. Because honestly, nobody would agree to have sex with someone who tells them upfront they have HPV.”

**# Discussion : The order of the reference numbers throughout the text is incorrect.**

Response : Thank you for noting this issue. In the revised manuscript, we have carefully reviewed and corrected the order of all in-text citation numbers to ensure sequential ordering consistent with the Vancouver citation style required by PLOS ONE. In the previous version, the reference list was not numbered in the order of first appearance in the text, resulting in several out-of-sequence citations throughout the manuscript—particularly in the Introduction and Discussion sections. In the revised version, all references have been renumbered according to their first appearance in the text. For example, references previously cited as [13–15] (stigma frameworks: Stangl et al., Lucas & Phelan, Goffman) have been renumbered to [20–21] to reflect their actual order of appearance following the addition of new references in the Introduction. All corresponding in-text citations and the reference list have been updated accordingly. We have also cross-checked all in-text citations against the reference list to confirm that the citation numbers and reference entries are fully consistent throughout the manuscript.

**# In the line 264, the source is not provided in Vancouver.**

Response : We appreciate the reviewer’s attention to detail. The reference at line 264 has been corrected to follow Vancouver formatting.

**Reviewer 2’s Comments:**

**Thank you for the opportunity to review this manuscript. As a reviewer for high-impact journals, I have assessed this study titled "Understanding barriers and facilitators to cervical cancer prevention among young Korean women" While the study addresses a critical public health issue the low screening rates among women in their 20s there are several areas regarding methodology and data representation that require attention to meet the rigorous standards of a top-tier journal.**

**#Abstract : The term “incompliance” appears to be a typo: it should likely be “in compliance”**

Response : We thank the reviewer for identifying this typographical error. The term has been corrected to “in compliance” in the revised manuscript.

**#Please specify the total duration of the study period or the specific dates of the interviews within the abstract to provide a temporal context immediately.**

Response : We appreciate this suggestion. The interview date has been added to the Methods sentence in the abstract. The revised sentence now reads : “Focus group interviews were conducted with 14 Korean women aged 20-29 years, divided into screeners and non-screener groups, on November 23, 2024.”

**#Introduction : You mention the screening rate for women in their 20s is 45.8%. It would be beneficial to briefly compare this to the rates in the 35-45 age bracket mentioned in the WHO strategy to further highlight the “gap” you are investigating.**

Response : We appreciate this valuable suggestion. A comparison sentence has been added to the Introduction to highlight the screening gap. The revised passage now reads : “However, the cervical cancer screening rate among Korean women in their 20s remains low at 45.8% as of 2022, substantially below WHO’s recommended 70-90% coverage target for women aged 35-45 years in cervical cancer elimination strategies.(World Health Organization,2022)” This comparison more clearly underscores the rationale for focusing on younger women in this study.

**#Method : The total N=14 is acceptable for qualitative research. But the imbalance between Group A (n=4) and Group B(n=10) is a concern. You state that transcript reviews continued until no new codes emerged. However, with only 4 participants in the “Screener” group, true thematic saturation for that specific cohort is difficult to claim.**

**#Please justify why the screener group was significantly smaller. Was this due to the low screening rate mentioned in the inro(45.8)? If so, explicitly state this as a recruitment challenge.**

Response : We appreciate the reviewer’s comment. The imbalance in group size was both a reflection of real-world screening patterns and a deliberate methodological consideration. As noted in the introduction, the cervical cancer screening rate among women in their 20s in Korea is relatively low(approximately 45.8%), meaning that individuals with prior screening experience constitute a smaller proportion of the target population. Consequently, Group A (screeners) was intentionally designed with a smaller sample size to reflect this underlying population distribution, while screening experience proved challenging in practice compared to non-screeners, which further contributed to the difference in group sizes.

**#Mention if any member checking(returning results to participants for validation) was performed to enhance the study’s credibility.**

Response : We appreciate the reviewer’s suggestion. Formal member checking—such as returning summarized transcripts or interpretations to participants for post-interview validation—was not conducted in this study, which may be considered a limitation. However, to enhance the credibility of the findings, a rigorous verification process was embedded within the focus group interviews. Specifically, at the end of each question–response exchange, the moderator consistently summarized participants’ responses and asked for confirmation to ensure that their intended meanings were accurately understood. This iterative process of real-time clarification and validation was applied throughout all sessions, thereby supporting the trustworthiness of the data.

**#Results : In Category 2, you mention a preference for KakaoTalk notifications. This is a vital “practical facilitator.” Ensure this is highlighted as a specific policy recommendation in your conclusion.**

Response : We thank the reviewer for this insightful comment. The Conclusion has been strengthened accordingly. The following sentence has been added: “In particular, transitioning from mail-based screening notifications to direct digital channels – such as KakaoTalk or text messaging – may serve as a practical and cost-effective strategy to improve screening reach among young women, for whom postal notifications are often missed due to frequent address changes or family interception.”

**#The quote for Sub-theme 3 is attributed to “Non-screener A” in the test, but the table should ensure all participant identifiers consistently match the demographic data provided.**

Response : We appreciate the reviewer for identifying this issue. The participant identifiers have been carefully reviewed and corrected to ensure consistency with the demographic data. Specifically, in line 211, “Screener B” has been updated to include the birth year (born ’02), and in line 216, “Screener D” has been updated to include the birth year (born ’99). These revisions ensure that all participant identifiers are consistently aligned with the demographic information presented in Table 2.

**#Discussion : The discussion effectively links the findings to broader social theories, such as Goffman’s “discrediting attribute” and structural health inequalities. You note that HPV vaccine cost is a major deterrent for those without fixed incomes. This is a strong point. I suggest adding a sentence on how “medical tourism” or “private clinic pricing” in Korea might further complicate this accessibility.**

Response : We appreciate this thoughtful suggestion. While this issue of private clinic pricing was not directly raised in our interview data, the following sentence has been added to the Discussion: “Furthermore, the pricing disparity between public health centers and private clinics in Korea may further complicate vaccine accessibility; women who are ineligible for the national immunization program are often redirected to private clinics where out-of-pocket costs are substantially higher, creating a two-tiered system in which access to prevention is inequitably distributed by income.”

**#The mention of expanding policies to men is insightful but feels slightly tangential to your data(which focused on women). Briefly connect this to how “de-gendering” the stigma might help the women in your study.**

Response : We thank the reviewer for this helpful suggestion. The Discussion has been revised to more explicitly connect gender-inclusive HPV prevention policies to stigma reduction for women. The following sentences have been added: “Importantly, including men in HPV prevention campaigns may also serve to de-gender the stigma surrounding cervical cancer. As reflected in the experience of participants in this study, much of the shame and reluctance associated with screening stems from the perception that HPV prevention is framed as a shared responsibility across genders rather than a female-specific burden, this may reduce the moral judgment directed at women and lower the psychological barriers to screening and vaccination identified in our data.”

**#The manuscript is generally intelligible, but there are minor typographical errors (e.g., “socal” instead of “social”, “participnats” instead of “participants”).**

Response : The manuscript has been carefully proofread, and identified typographical errors have been corrected throughout the text. In addition, the manuscript has undergone professional English language editing to ensure overall linguistic accuracy and clarity.

**#Ensure all citations in the text (e.g., [13-15]) correspond exactly to the numbered list in the References section.**

Response : We thank the reviewer for this important observation. We have conducted a comprehensive cross-check of all in-text citations against the reference list in the revised manuscript. The following corrections and updates were made: First, following the addition of new references in the Introduction — specifically Farajimakin (2024) [18] and Shpendi et al. (2025) [19] — and the restructuring of the reference list, all subsequent reference numbers were systematically incremented to maintain sequential order. As a result, the reference list in the revised manuscript consists of 39 references in total. Second, three incomplete citations that appeared in the previous version as "[15, ]", "[31, ]", and "[35, ]" — indicating placeholder numbers that had not been assigned — have been fully resolved. Specifically:

- "[15, ]" (FGI methodology) has been corrected to [16] (Kim H, et al., 2004)
- "[31, ]" (cervical cancer stigma at multiple levels) has been corrected to [31, 36] (Morse et al., 2023; Link & Phelan, 2001)
- "[35, ]" (neutral sexual health education) has been corrected to [34] (Albert Sekhar et al., 2024)

Third, the citation for the large-scale meta-analysis on expanding HPV vaccination to men has been updated from the previous "[34, 35]" to [38, 39] (Harder et al., 2018; Drolet et al., 2019) to reflect the new reference numbering. All in-text citations have been verified to correspond sequentially and accurately to the numbered reference list, consistent with the Vancouver citation style required by PLOS ONE.
