# Supplementary material for: Understanding barriers and facilitators to cervical cancer prevention among young Korean Women: A focus group interview study
Source: PLoS One. 2026 Jul 10;21(7):e0353242. doi: 10.1371/journal.pone.0353242 (PMC13353974; doi:10.1371/journal.pone.0353242)
Supplement: Peer review history

# Peer review: Decision Letter 0

**DOI:** 10.1371/journal.pone.0353242.r001

**Authors:** Fatemeh Zarei

23 Feb 2026

PONE-D-25-59809

Understanding barriers and facilitators to cervical cancer prevention among young Korean Women: A focus group interview study

PLOS One

Dear Dr. Mina Suh

Thank you for submitting your manuscript to PLOS ONE. After careful consideration, we feel that it has merit but does not fully meet PLOS ONE’s publication criteria as it currently stands. Therefore, we invite you to submit a revised version of the manuscript that addresses the points raised during the review process.

==============================

Dear Authors,

Thank you for submitting your manuscript, **"Understanding barriers and facilitators to cervical cancer prevention among young Korean women,"**  to our journal.

After reviewing your submission and the reports from reviewers, I am pleased to invite you to revise your manuscript. Both reviewers see the value in your investigation of the 45.8% screening rate "gap," particularly within the Korean socio-cultural context. However, significant revisions regarding methodological transparency, data representation, and citation accuracy are required before a final decision can be reached. Please provide a **Point-by-Point Response**  to the reviewers' comments when you submit your revised files.

==============================

Please submit your revised manuscript by Apr 09 2026 11:59PM. If you will need more time than this to complete your revisions, please reply to this message or contact the journal office at plosone@plos.org. When you're ready to submit your revision, log on to https://www.editorialmanager.com/pone/ and select the 'Submissions Needing Revision' folder to locate your manuscript file.

Please include the following items when submitting your revised manuscript:

- A letter that responds to each point raised by the academic editor and reviewer(s). You should upload this letter as a separate file labeled 'Response to Reviewers'.
- A marked-up copy of your manuscript that highlights changes made to the original version. You should upload this as a separate file labeled 'Revised Manuscript with Track Changes'.
- An unmarked version of your revised paper without tracked changes. You should upload this as a separate file labeled 'Manuscript'.

If you would like to make changes to your financial disclosure, please include your updated statement in your cover letter. Guidelines for resubmitting your figure files are available below the reviewer comments at the end of this letter.

If applicable, we recommend that you deposit your laboratory protocols in protocols.io to enhance the reproducibility of your results. Protocols.io assigns your protocol its own identifier (DOI) so that it can be cited independently in the future. For instructions see: https://journals.plos.org/plosone/s/submission-guidelines#loc-laboratory-protocols. Additionally, PLOS ONE offers an option for publishing peer-reviewed Lab Protocol articles, which describe protocols hosted on protocols.io. Read more information on sharing protocols at https://plos.org/protocols?utm_medium=editorial-email&utm_source=authorletters&utm_campaign=protocols.

We look forward to receiving your revised manuscript.

Kind regards,

Fatemeh Zarei, PhD

Academic Editor

PLOS One

Journal Requirements:

When submitting your revision, we need you to address these additional requirements.

1. Please ensure that your manuscript meets PLOS ONE's style requirements, including those for file naming. The PLOS ONE style templates can be found at

https://journals.plos.org/plosone/s/file?id=wjVg/PLOSOne_formatting_sample_main_body.pdf and

https://journals.plos.org/plosone/s/file?id=ba62/PLOSOne_formatting_sample_title_authors_affiliations.pdf.

2. We note that you have indicated that there are restrictions to data sharing for this study. For studies involving human research participant data or other sensitive data, we encourage authors to share de-identified or anonymized data. However, when data cannot be publicly shared for ethical reasons, we allow authors to make their data sets available upon request. For information on unacceptable data access restrictions, please see http://journals.plos.org/plosone/s/data-availability#loc-unacceptable-data-access-restrictions.

Before we proceed with your manuscript, please address the following prompts:

a) If there are ethical or legal restrictions on sharing a de-identified data set, please explain them in detail (e.g., data contain potentially identifying or sensitive patient information, data are owned by a third-party organization, etc.) and who has imposed them (e.g., a Research Ethics Committee or Institutional Review Board, etc.). Please also provide contact information for a data access committee, ethics committee, or other institutional body to which data requests may be sent.

b) If there are no restrictions, please upload the minimal anonymized data set necessary to replicate your study findings to a stable, public repository and provide us with the relevant URLs, DOIs, or accession numbers. Please see http://www.bmj.com/content/340/bmj.c181.long for guidelines on how to de-identify and prepare clinical data for publication. For a list of recommended repositories, please see https://journals.plos.org/plosone/s/recommended-repositories. You also have the option of uploading the data as Supporting Information files, but we would recommend depositing data directly to a data repository if possible.

Please update your Data Availability statement in the submission form accordingly.

3. We note that the grant information you provided in the ‘Funding Information’ and ‘Financial Disclosure’ sections do not match.

When you resubmit, please ensure that you provide the correct grant numbers for the awards you received for your study in the ‘Funding Information’ section.

4. Thank you for stating the following in the Acknowledgments Section of your manuscript:

“This study was supported by a Grant-in-Aid for Cancer Research and Control from the National Cancer Center, Korea.(grant number : 24H1071-2)”

We note that you have provided additional information within the Acknowledgements Section that is not currently declared in your Funding Statement. Please note that funding information should not appear in the Acknowledgments section or other areas of your manuscript. We will only publish funding information present in the Funding Statement section of the online submission form.

Please remove any funding-related text from the manuscript and let us know how you would like to update your Funding Statement. Currently, your Funding Statement reads as follows:

“The author(s) received no specific funding for this work.”

Please include your amended statements within your cover letter; we will change the online submission form on your behalf.

5. If the reviewer comments include a recommendation to cite specific previously published works, please review and evaluate these publications to determine whether they are relevant and should be cited. There is no requirement to cite these works unless the editor has indicated otherwise.

[Note: HTML markup is below. Please do not edit.]

Reviewers' comments:

Reviewer's Responses to Questions

**Comments to the Author**

1. Is the manuscript technically sound, and do the data support the conclusions?

The manuscript must describe a technically sound piece of scientific research with data that supports the conclusions. Experiments must have been conducted rigorously, with appropriate controls, replication, and sample sizes. The conclusions must be drawn appropriately based on the data presented.

Reviewer #1: Yes

Reviewer #2: Yes

**********

2. Has the statistical analysis been performed appropriately and rigorously?

Reviewer #1: Yes

Reviewer #2: I Don't Know

**********

3. Have the authors made all data underlying the findings in their manuscript fully available?

The PLOS Data policy requires authors to make all data underlying the findings described in their manuscript fully available without restriction, with rare exception (please refer to the Data Availability Statement in the manuscript PDF file). The data should be provided as part of the manuscript or its supporting information, or deposited to a public repository. For example, in addition to summary statistics, the data points behind means, medians and variance measures should be available. If there are restrictions on publicly sharing data—e.g. participant privacy or use of data from a third party—those must be specified.

Reviewer #1: Yes

Reviewer #2: Yes

**********

4. Is the manuscript presented in an intelligible fashion and written in standard English?

PLOS ONE does not copyedit accepted manuscripts, so the language in submitted articles must be clear, correct, and unambiguous. Any typographical or grammatical errors should be corrected at revision, so please note any specific errors here.

Reviewer #1: Yes

Reviewer #2: Yes

**********

5. Review Comments to the Author

Please use the space provided to explain your answers to the questions above. You may also include additional comments for the author, including concerns about dual publication, research ethics, or publication ethics. (Please upload your review as an attachment if it exceeds 20,000 characters)

Reviewer #1: Introduction

A paragraph should not be less than 5 lines.

The epidemiology is old. It must be for the last two years.

On line 62, the source must be cited.

On line 68, after the years, the source must be cited.

On line 69, after the efforts, the source must be cited.

The introduction should include a background of recent research.

Method

"Who do you think is likely to get cervical cancer?"/ This sentence should be corrected.

Results

More quotes should be provided in the findings section. Because there is a need for more evidence for subthemes for the readers.

Discussion

The order of the reference numbers throughout the text is incorrect.

In line 264, the source is not provided in Vancouver.

Reviewer #2: Thank you for the opportunity to review this manuscript. As a reviewer for high-impact journals, I have assessed this study titled "Understanding barriers and facilitators to cervical cancer prevention among young Korean women"

While the study addresses a critical public health issue the low screening rates among women in their 20s there are several areas regarding methodology and data representation that require attention to meet the rigorous standards of a top-tier journal.

1. Abstract

The term "incompliance" appears to be a typo; it should likely be "in compliance."

Please specify the total duration of the study period or the specific dates of the interviews within the abstract to provide a temporal context immediately.

2. Introduction

You mention the screening rate for women in their 20s is 45.8%. It would be beneficial to briefly compare this to the rates in the 35-45 age bracket mentioned in the WHO strategy to further highlight the "gap" you are investigating.

3. Methods

• The total N=14 is acceptable for qualitative research, but the imbalance between Group A (n=4) and Group B (n=10) is a concern. You state that transcript reviews continued until no new codes emerged. However, with only 4 participants in the "Screener" group, true thematic saturation for that specific cohort is difficult to claim.

• Please justify why the screener group was significantly smaller. Was this due to the low screening rate mentioned in the intro (45.8%)? If so, explicitly state this as a recruitment challenge .

• Mention if any member checking (returning results to participants for validation) was performed to enhance the study's credibility.

4. Results

• In Category 2, you mention a preference for KakaoTalk notifications. This is a vital "practical facilitator." Ensure this is highlighted as a specific policy recommendation in your conclusion.

• The quote for Sub-theme 3 is attributed to "Non-screener A" in the text, but the table should ensure all participant identifiers consistently match the demographic data provided.

5. Discussion

• The discussion effectively links the findings to broader social theories, such as Goffman’s "discrediting attribute" and structural health inequalities.

• You note that the HPV vaccine cost is a major deterrent for those without fixed incomes. This is a strong point. I suggest adding a sentence on how "medical tourism" or "private clinic pricing" in Korea might further complicate this accessibility.

• The mention of expanding policies to men is insightful but feels slightly tangential to your data (which focused on women). Briefly connect this to how "de-gendering" the stigma might help the women in your study.

The manuscript is generally intelligible, but there are minor typographical errors (e.g., "socal" instead of "social" , "participnats" instead of "participants" ).

Ensure all citations in the text (e.g., [13-15]) correspond exactly to the numbered list in the References section.

**********

6. PLOS authors have the option to publish the peer review history of their article (what does this mean?). If published, this will include your full peer review and any attached files.

If you choose “no”, your identity will remain anonymous but your review may still be made public.

**Do you want your identity to be public for this peer review?**  For information about this choice, including consent withdrawal, please see our Privacy Policy.

Reviewer #1: **Yes:**  Fatteme Raiisi

Reviewer #2: **Yes:**  Fatemeh Zarei

**********

[NOTE: If reviewer comments were submitted as an attachment file, they will be attached to this email and accessible via the submission site. Please log into your account, locate the manuscript record, and check for the action link "View Attachments". If this link does not appear, there are no attachment files.]

To ensure your figures meet our technical requirements, please review our figure guidelines: https://journals.plos.org/plosone/s/figures

You may also use PLOS’s free figure tool, NAAS, to help you prepare publication quality figures: https://journals.plos.org/plosone/s/figures#loc-tools-for-figure-preparation.

NAAS will assess whether your figures meet our technical requirements by comparing each figure against our figure specifications.

# Author comment: Author response to Decision Letter 1

**DOI:** 10.1371/journal.pone.0353242.r002

4 Jun 2026

Dear Editors,

Please find our detailed responses to the reviewers’ comments in the attached “Response to Reviewers” document. We have revised the manuscript accordingly and addressed all comments carefully.

Thank you very much for your consideration.

Sincerely,

Suh, Mina

Attachment Submitted filename: Response to Reviwers.docx

# Peer review: Decision Letter 1

**DOI:** 10.1371/journal.pone.0353242.r003

**Authors:** Fatemeh Zarei

22 Jun 2026

Understanding barriers and facilitators to cervical cancer prevention among young Korean Women: A focus group interview study

PONE-D-25-59809R1

Dear Dr. Mina Suh

We’re pleased to inform you that your manuscript has been judged scientifically suitable for publication and will be formally accepted for publication once it meets all outstanding technical requirements.

Within one week, you’ll receive an e-mail detailing the required amendments. When these have been addressed, you’ll receive a formal acceptance letter and your manuscript will be scheduled for publication.

An invoice will be generated when your article is formally accepted. Please note, if your institution has a publishing partnership with PLOS and your article meets the relevant criteria, all or part of your publication costs will be covered. Please make sure your user information is up-to-date by logging into Editorial Manager at Editorial Manager® and clicking the ‘Update My Information' link at the top of the page. For questions related to billing, please contact billing support.

If your institution or institutions have a press office, please notify them about your upcoming paper to help maximize its impact. If they’ll be preparing press materials, please inform our press team as soon as possible -- no later than 48 hours after receiving the formal acceptance. Your manuscript will remain under strict press embargo until 2 pm Eastern Time on the date of publication. For more information, please contact onepress@plos.org.

Kind regards,

Fatemeh Zarei, PhD

Academic Editor

PLOS One

Additional Editor Comments (optional):

Reviewers' comments:

Reviewer's Responses to Questions

**Comments to the Author**

1. If the authors have adequately addressed your comments raised in a previous round of review and you feel that this manuscript is now acceptable for publication, you may indicate that here to bypass the “Comments to the Author” section, enter your conflict of interest statement in the “Confidential to Editor” section, and submit your "Accept" recommendation.

Reviewer #1: All comments have been addressed

Reviewer #2: (No Response)

**********

2. Is the manuscript technically sound, and do the data support the conclusions?

The manuscript must describe a technically sound piece of scientific research with data that supports the conclusions. Experiments must have been conducted rigorously, with appropriate controls, replication, and sample sizes. The conclusions must be drawn appropriately based on the data presented.

Reviewer #1: Yes

Reviewer #2: Yes

**********

3. Has the statistical analysis been performed appropriately and rigorously?

Reviewer #1: I Don't Know

Reviewer #2: Yes

**********

4. Have the authors made all data underlying the findings in their manuscript fully available?

The PLOS Data policy requires authors to make all data underlying the findings described in their manuscript fully available without restriction, with rare exception (please refer to the Data Availability Statement in the manuscript PDF file). The data should be provided as part of the manuscript or its supporting information, or deposited to a public repository. For example, in addition to summary statistics, the data points behind means, medians and variance measures should be available. If there are restrictions on publicly sharing data—e.g. participant privacy or use of data from a third party—those must be specified.

Reviewer #1: Yes

Reviewer #2: Yes

**********

5. Is the manuscript presented in an intelligible fashion and written in standard English?

PLOS ONE does not copyedit accepted manuscripts, so the language in submitted articles must be clear, correct, and unambiguous. Any typographical or grammatical errors should be corrected at revision, so please note any specific errors here.

Reviewer #1: Yes

Reviewer #2: (No Response)

**********

6. Review Comments to the Author

Please use the space provided to explain your answers to the questions above. You may also include additional comments for the author, including concerns about dual publication, research ethics, or publication ethics. (Please upload your review as an attachment if it exceeds 20,000 characters)

Reviewer #1: All items have been corrected. The author has taken all the points into account and explained them well at the end of the article. It has been added to the quality and validity of the qualitative study.

Reviewer #2: No more comments left Thanks for efforts ,

**********

7. PLOS authors have the option to publish the peer review history of their article (what does this mean?). If published, this will include your full peer review and any attached files.

If you choose “no”, your identity will remain anonymous but your review may still be made public.

**Do you want your identity to be public for this peer review?**  For information about this choice, including consent withdrawal, please see our Privacy Policy.

Reviewer #1: **Yes:**  Fatteme Raiisi

Reviewer #2: **Yes:**  Fatemeh Zarei

**********

# Editor report: Acceptance letter

**DOI:** 10.1371/journal.pone.0353242.r004

**Authors:** Fatemeh Zarei

PONE-D-25-59809R1

PLOS One

Dear Dr. Suh,

I'm pleased to inform you that your manuscript has been deemed suitable for publication in PLOS One. Congratulations! Your manuscript is now being handed over to our production team.

At this stage, our production department will prepare your paper for publication. This includes ensuring the following:

* All references, tables, and figures are properly cited

* All relevant supporting information is included in the manuscript submission,

* There are no issues that prevent the paper from being properly typeset

You will receive further instructions from the production team, including instructions on how to review your proof when it is ready. Please keep in mind that we are working through a large volume of accepted articles, so please give us a few days to review your paper and let you know the next and final steps.

Lastly, if your institution or institutions have a press office, please let them know about your upcoming paper now to help maximize its impact. If they'll be preparing press materials, please inform our press team within the next 48 hours. Your manuscript will remain under strict press embargo until 2 pm Eastern Time on the date of publication. For more information, please contact onepress@plos.org.

You will receive an invoice from PLOS for your publication fee after your manuscript has reached the completed accept phase. If you receive an email requesting payment before acceptance or for any other service, this may be a phishing scheme. Learn how to identify phishing emails and protect your accounts at https://explore.plos.org/phishing.

If we can help with anything else, please email us at customercare@plos.org.

Thank you for submitting your work to PLOS ONE and supporting open access.

Kind regards,

PLOS ONE Editorial Office Staff

on behalf of

Dr. Fatemeh Zarei

Academic Editor

PLOS One
